# On the Uses of Turpentine

**Published:** 1854-02

**Authors:** Samuel Thompson

**Affiliations:** Albion, Illinois


#### THE NORTH-WESTERN
### MEDICAL AND SURGICAL JOURNAL.

NEW SERIES.

VOL. III. FEBRUARY, 1854. NO. 2.

### ORIGINAL COMMUNICATIONS.

*Transactions of the Illinois State Medical Society,§ for the year* 1853. "On the Uses of Turpentine; by Samuel Thompson, M.D., of Albion, Illinois.

*A Dictionary of Practical Medicine*, Comprising General Pathology, the Nature and Treatment of Diseases, Morbid Strictures, &c: by James Copland, M. D., F. R. S. Re-published by Harper & Brothers, New York.

"RENDER UNTO CÆSAR THE THINGS THAT ARE CÆSAR S."

(*Continued from last Number.*)

Dr. Thompson refers to one case of amaurosis in Braithwaite's Retrospect, January, 1852:

"The success which has resulted from the exhibition of the oil of turpentine in iritis, induced me to prescribe it, after depletions, in two cases of this form of amaurosis; (amaurosis from inflammation of the retina and internal parts of the eye,) and with satisfactory results in both. In persons far advanced in life, in scrofulous subjects, and in debilitated persons, this oil is certainly a less hazardous medicine than the mercury exhibited so as to effect the system." Copland op. cit., vol. 1, page 6: 1831. We have no experience in the use of the remedy in this disease.

Before we close our observations on the applicability of Turpentine to ophthalmic diseases it may not be altogether irrelevant to place on record a few cases of chronic purulent ophthalmia cured by the direct application of the oil of Turpentine to the inflamed mucous membrane. The cases were particularly obstinate and resisted all our efforts to cure. After the lapse of a few weeks from making our last prescription we met one of the worst cases perfectly cured, and before asking any questions had inwardly rejoiced at the full success of our last remedy; but we were quickly enlighted on the matter by the patient telling us that he had cured himself by the use of Turpentine. "Did it not make your eyes smart badly?" we asked "Smart, why yes--like h--ll--but what of that—in an hour I could open my eyes, and keep them open, and in four or five days were as you now see them." He then told me that the other cases in the neighbourhood were all cured by the same means, and that the man who advised him to use it said that it cured all the cases (*I think*, in Ohio,) that the Drs. could not. Briggs, who translated Scarpa's celebrated work, recommended the application of a few drops of Turpentine to the anurous membrane when the discharge was profuse. We have not tried it and very much question whether the patients would submit to it, if recommended or applied by a Physician; but at the same time, judging from analogy, we should have no doubt of its utility in this class of diseases.

We think, that had Dr. Thompson searched a little more, instead of finding but one author who had recommended turpentine for tænia—he would have had some little difficulty in finding one, who has not advised the use of it, and who has alluded to the subject, at least amongst writers whose works are not over thirty years old. When we commenced the study of medicine, thirty-four years ago, Dr. Thomas (the modern practice of physic,) was the author, whose opinions on medical subjects generally guided the mass of British practitioners, and he refers to it as being *recently* used and recommended in cases of tænia; he is now laid upon the shelf, but every subsequent writer that we know of, alludes to the same remedy for the same worm We well recollect seeing two ounces given to a man suspected of being troubled with a tapeworm in the year 1823 or 1824, in a public institution. The patient first became talkative and merry, and he left the dispensary in a state somewhat similar to intoxication. He returned with a dozen oranges, which he gave to the other apprentice, and on his hesitating to receive them, was immediately knocked down. We had some difficulty in calming the man, and the next day he stoutly denied having any knowledge of the affray. So far as the worm was concerned, it had no effect, it is possible there was not any there, for the physicians were mere theorists, and appeared to us to care more about experimenting with new remedies than judiciously using old ones. In connection with this subject, we clearly recollect reading somewhere, that turpentine had been used for tænia long before the period that Thomas published his work, but had fallen into disuse, until, some one took it experimentally and dislodged the worm, but the article, probably containing nothing practically useful, is not noticed in our general index, and consequently we cannot refer the reader to it.

Hemorrhage is the next disease alluded to by Dr. Thompson, and one of the most important in the list, and, when it occurs under certain conditions, is, in our opinion, more easily and certainly arrested by turpentine, than by any other remedy, or combination of remedies. Of course, we do not intend to claim for Dr. Copland priority of use as a styptic, but this article on Hemorrhage was published long before Mr. Vincent gave his opinion to the world, and referred to by Dr. Thompson, or Dr. Budd delivered his lecture, published in the *Medical Times* and reprinted, in part, in the *American Journal of Medical Sciencies*, for October, 1850, page 473; but overlooked by Dr. Thompson, and to which we here refer our readers. It is a good article in the main; the opinion of Hunter, Vincent, and the author is given, but no reference to Copland: with the following, however, we cannot agree. "In what are called active hemorrhages, it (turpentine) would not seem to be so suitable a medicine. We must not, however, be hampered by definitions like these in cases where life is threatened. Cases of hemorrhage are cases in which, beyond all others, the nice distinctions of pathology have to give way before the great emergencies of practice." Now it is the recognition of these "nice distinctions of pathology," and *practice based upon them*, that, in many instances, makes one medical man superior to another: and, when these "nice distinctions give way before the great emergencies of practice," practice at once ceases to be scientific, becomes empirical, and not unfrequently injurious. Discarding these "nice distinctions," there can be little doubt, was the chief reason why turpentine was so little used in the last, and early part of the present century, and had lost that character in this class of diseases it once possessed. And (here we have to differ somewhat from Dr. Copland) if any one expects to find more benefit from turpentine than from other remedies, in the purely active or sthenic forms of hemorrhage, he will be disappointed—or his experience will not be in harmony with ours. It is in the purely *asthenic* kind—where there is not only an atonic state, a loss of vital cohesion of the capillaries and venous conjestion, but also an altered and deteriorated condition of the blood, with diminished vital power and generally increased vascular action, that turpentine will be found not only useful, but in a great majority of cases, the best remedy we can employ. The following quotation is from Copland's article on the pathology of hemorrhage and its treatment without reference to its seat, and first in regard to his opinion on "nice distinctions of pathology." "*Hemorrhages from changes in vital power and vascular action* require the utmost pathological discrimination and practical decision. Upon the opinion that will be formed as to the degrees of augmented action, or of diminished power, or of vascular repletion, or of asthenia, not only will the success of the treatment, but also the life of the patients depend. And amongst the most difficult of the many difficult topics with which the practical physician will have to concern himself, none is more difficult or more important, than to discriminate the pathological conditions just mentioned.

"The *spirit of turpentine* appears to have been employed by the ancients in the treatment of hemorrhages. It was much used, both internally and externally, during the sixteenth century, but had afterwards fallen into disuse. In the year 1817 I employed it internally in these diseases, and have since continued to prescribe it. (See my *Memoir on the use of Terebinthinate Remedies in Disease, London Medical and Physical Journal* for July and August, 1821.) It constringes the capillaries of the part to which it is applied; but, owing to its stimulating action on the nerves, sthenic vascular reaction frequently follows; which, however, soon subsides. When used in large quantity, these effects are proportionately great; and it thereby exerts a powerful derivative influence. When absorbed into the circulation, its astringent effect on the capillaries is also remarkable. Its action varies much with the dose, relatively to the vital energy of the patient. When the dose is large, it reduces the strength and frequency of the heart's action, especially when they are much increased; and hence it is an appropriate remedy in the more active forms of hemorrhage, in as much as, with its constringing action on the capillaries, it weakens the *vis a tergo.* When given in smaller doses, and carried into the blood, it increases the tone and changes or modifies the action of the extreme vessels. From a very extensive experience of this medicine in hemorrhages and other diseases, I may add, that large doses of it should be prescribed with caution, when the powers of life are very much depressed; and that, when a considerable dose of it has been given in such cases, it ought to be carried off by stool. The existence of inflammatory action does not contra-indicate its use, as many have supposed from a misconception of its operation; for it lowers vascular excitement, and prevents effusion and the formation of coagulable lymph, especially when taken in sufficiently large and repeated doses. When the powers of life are much impaired, and after copious evacuations of blood, small and frequent doses of it only ought to be given conjoined with tonics, restoratives etc."

We well remember reading the *Memoir* of Dr. Copland when it came out in the *Journal;* and since that time have used turpentine, and seen it used by others, in every form of asthenic hemorrhage with the happiest effect. In cases of profuse menstruation with anemic countenances and lax fibre, we have given it for a few days before the expected period with invariable success; and these cases are too common from some cause or other in this State. Of course we use chalybeates and tonics at all times, with such other adjuvants as each case may demand. Dr. Thompson makes no allusion to any effect of turpentine on the nervous system, although he must have witnessed it repeatedly. There are few physicians, that have not witnessed the change of countenance that occurs instaneously when the system receives an injury from which it cannot recover, although many of these accidents are trifling so far as suffering is concerned at least in the beginning, yet the vital powers are aware that irreparable mischief has been done, and the shock is manifest to the most careless observer. We have again and again seen an opposite effect produced on the countenance from the action of turpentine on the nervous system, when it has been appropriately prescribed in extreme cases of disease: and this effect has been produced frequently long before the medicine could possibly be absorbed, and through the circulation reach the disease. The vital powers appear to be aware that the disease can be overcome, that they have received the required assistance from the remedy, and not only the expression of the countenance, but the aspect of the case is immediately changed for the better: and we have observed this rapid improvement more frequently from the exhibition of turpentine than from any other remedy.

(*To be continued.*)

